# Extracellular matrix-mimic nanofibrous microspheres: fabrication and application in stem cell delivery for tissue repair and regeneration

**DOI:** 10.1016/j.mtbio.2026.103381

**Published:** 2026-06-21

**Authors:** Tengfei Tian, Jiashan Zhang, Jiahui Wu, Chuanfeng An, Huanan Wang, Yonggang Zhang

**Affiliations:** a Department of Orthopedics, Central Hospital of Dalian University of Technology, Dalian, Liaoning, 116024, PR China; b MOE Key Laboratory of Bio-Intelligent Manufacturing, Dalian Key Laboratory of Artificial Organ and Regenerative Medicine, School of Bioengineering, Dalian University of Technology, Dalian, Liaoning, 116024, PR China; c State Key Laboratory of Fine Chemicals, Dalian University of Technology, Dalian, Liaoning, 116024, PR China; d Ophthalmology and Transformational Innovation Research Center, Dalian Third People's Hospital Affiliated to Dalian University of Technology, Dalian, Liaoning, 116033, PR China

**Keywords:** Nanofibrous microsphere, Stem cell, Cell carrier, Tissue engineering

## Abstract

Stem cells, featuring remarkable self-renewal ability, unique multi-differentiation potential, distinguished paracrine effect and strong immunomodulatory function, have emerged as a powerful and competitive candidate for treating severe diseases and injuries. Direct injection of stem cells always suffers from limited cell retention in target tissues. Growing evidence suggests that nanofibrous microspheres with natural extracellular matrix-like topography, interconnected pores, high porosity, high specific surface area, and distinct injectability may serve as promising stem cell carriers that facilitate cell attachment, spreading, proliferation, retention and expression of specific genes. Over the past few decades, tremendous efforts have been devoted to developing nanofibrous microspheres with diverse composition, size, morphology and structure by using a variety of fabrication techniques. In this review, we provide an overview of recent progress in the development of nanofibrous microspheres with a focus on their preparation method, chemical composition, physicochemical property, and bioactivity, and highlight challenges and perspectives for future research directions.

## Graphical abstract

**Figure:**
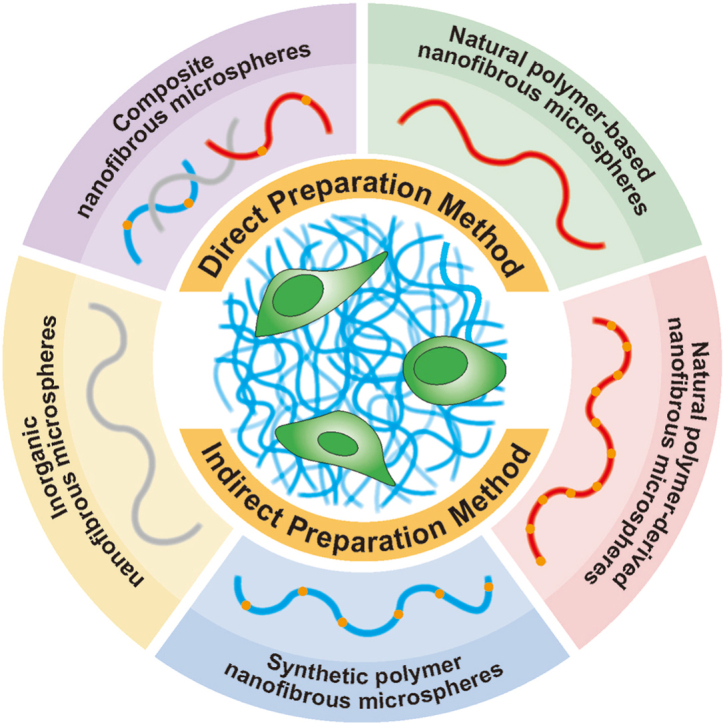

## 1 Introduction

Stem cells are undifferentiated or partially differentiated cells with remarkable self-renewal ability, unique multi-differentiation potential, distinguished paracrine effect and strong immunomodulatory function [1]. They can be broadly classified as either pluripotent (embryonic stem cells and induced pluripotent stem cells) or somatic (adult stem cells) depending on the differentiation potential [2]. For instance, as a typical type of adult stem cell, mesenchymal stem cells (MSCs) are able to differentiate into a variety of cell types, including adipocytes, osteoblasts, chondrocytes, and myocytes [3]. In addition, they could secrete a diversity of growth factors and cytokines, such as vascular endothelial growth factor (VEGF), transforming growth factor, and interleukins to modulate cell behavior [4]. Moreover, MSCs are capable of regulating immune responses, e.g., through suppressing T-cell proliferation, dendritic cell maturation, and B-cell activation [5]. All these characteristics make stem cells a powerful and competitive candidate for treating severe diseases and injuries, such as cancer, diabetes, leukemia, neurodegenerative diseases, immune diseases, and large-sized tissue damage that can't be effectively treated by conventional methods [[6], [7], [8]]. Stem cell therapy, a treatment using stem cells to facilitate tissue repair and disease management, has become a cutting-edge research area in tissue engineering and regenerative medicine over the past decade [9,10]. Unfortunately, clinical application of stem cells is extremely restricted by insufficient cell supply. The quantity of stem cells isolated from donors is hard to meet the effective dose required for clinical treatment. Furthermore, stem cell injection suffers from limited cell retention in target tissues, which inevitably results in constrained therapeutic effect [11]. Therefore, there remains a massive demand for developing strategies for ex-vivo production and efficient delivery of stem cells.

Conventional ex-vivo expansion of stem cell usually uses two-dimensional (2D) monolayer culture on tissue culture plates. Nevertheless, the cell expansion efficiency is low [12]. More importantly, stem cells experiencing long-term of 2D monolayer culture may lose their stemness due to the fact that 2D culture environment is quite different from in vivo cell growth microenvironment [13,14]. In biological tissues, cells are supported by extracellular matrix (ECM) with an intricate three-dimensional (3D) nanofibrous network [15]. ECM plays a crucial role in modulating cell behaviors including attachment, proliferation and differentiation [16]. Therefore, 3D cell culture systems which simulate the local microenvironment of cells have emerged and present an appealing alternative to 2D culture methods for ex-vivo stem cell expansion. Compared with 2D culture methods, 3D culture systems more accurately mimic the in vivo 3D microenvironment [16,17]. Biomimetic 3D microenvironment facilitates information exchange between cells and ECM, promotes stem cell self-renewal, and helps stem cells maintain their original biological characteristics [18]. 3D culture systems can be divided into scaffold-free and scaffold-based approaches. Scaffold-based 3D culture system refers to co-cultivation of cell carriers and stem cells. The cell carriers serve as scaffolds for stem cell attachment, migration and proliferation [[19], [20], [21], [22]]. So far, various kinds of cell carriers have been developed, such as film, hydrogel and sphere [[23], [24], [25], [26], [27]]. Compared with film and hydrogel [28], sphere carriers, more precisely, microsphere carriers are smaller in size, therefore possessing larger specific surface area for stem cell attachment, growth and migration. In addition, the small size endows these microsphere carriers with outstanding injectability, shape adaptability, and significant advantages in treating tissue defects with irregular shapes and minimally invasive treatment. According to their physical and chemical properties, microspheres can be divided into five types, namely, macroporous microspheres, hollow microspheres, core-shell microspheres, surface-modified microspheres, and nanofibrous microspheres [29]. Due to the presence of pores, hollow structures and specific chemicals, macroporous microspheres, hollow microspheres, core-shell microspheres, and surface-modified microspheres exhibit significant advantages as cell carriers in facilitating cell adhesion, infiltration, and differentiation. However, they can't provide cells with a three-dimensional network microenvironment similar to the natural ECM [30]. Notably, nanofibrous microspheres stand out from the rest of stem cell carriers owing to their distinctive advantages, i.e., high surface-to-volume ratio for cell colonization and expansion, cell-cell and cell-niche interaction, highly interconnected porous structure providing conducive environment for oxygen and nutrients supply and waste removal, fibrous topographical feature similar to that of natural ECM facilitating stem cells to maintain their stemness [29], small dimension suitable for minimally invasive surgery and complex defect treatment [31]. All these characteristics make nanofibrous microspheres an ideal candidate for expansion and delivery of stem cells.

**Fig. 1:**
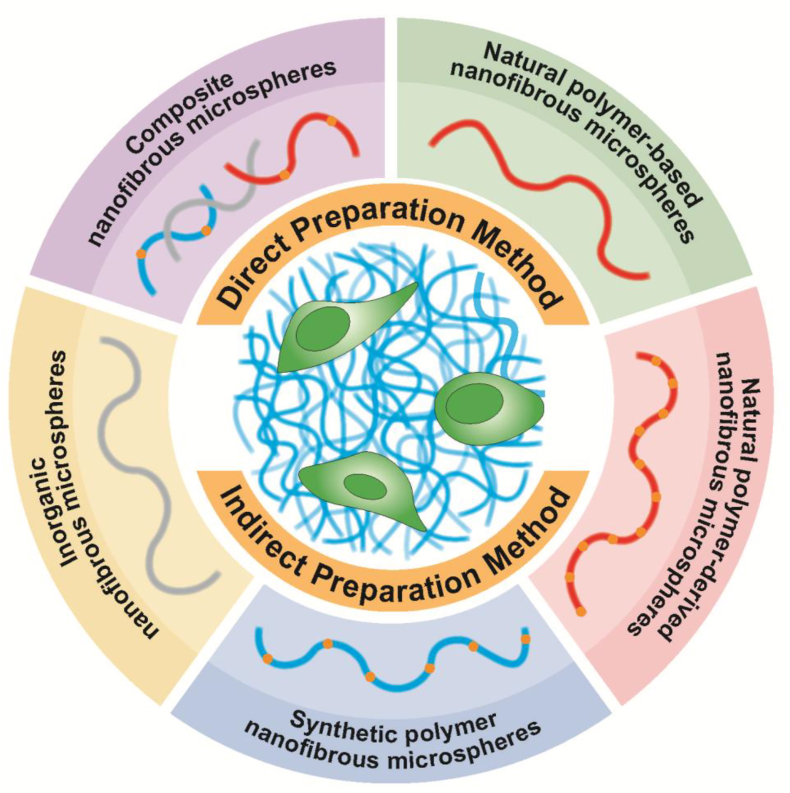
Nanofibrous microspheres made from natural and synthetic biomaterials for cell expansion, delivery and tissue regeneration.

To date, a variety of nanofibrous microspheres consisting of different kinds of biomaterials have been developed by using diverse fabrication methods, and extensive research efforts have been expended to achieve delicate control of their structure as well as decipher the interaction between stem cells and these nanofibrous microsphere [32]. Even though the clinical translation of nanofibrous microspheres-based stem cell therapy is yet to come, tremendous in vitro and in vivo data have proved the effectiveness of nanofibrous microsphere carriers in stem cell expansion, delivery and disease treatment, such as cartilage regeneration, myocardial repair, bone defect repair, and more [26,33]. In this review, we first provide a review of the state-of-the-art fabrication techniques of extracellular matrix-mimic nanofibrous microspheres (microspheres with nanofibrous topographical feature similar to that of the fibrous network of natural ECM). We then introduce the progress of nanofibrous microspheres made from a variety of biomaterials (Fig. 1) with a focus on their composition, structure and application as stem cell carriers for tissue repair and regeneration. Next, we discuss the underlying mechanisms of how the physical properties of nanofibrous microspheres influence stem cell behavior. Finally, we conclude by summarizing the content, analyzing existing challenges and offering an outlook on perspectives for future research directions. This review will provide a useful reference for the design of new, advanced nanofibrous microsphere biomaterials as stem cell carriers for tissue repair and regeneration application.

## 2 Fabrication techniques of nanofibrous microspheres

**Fig. 2:**
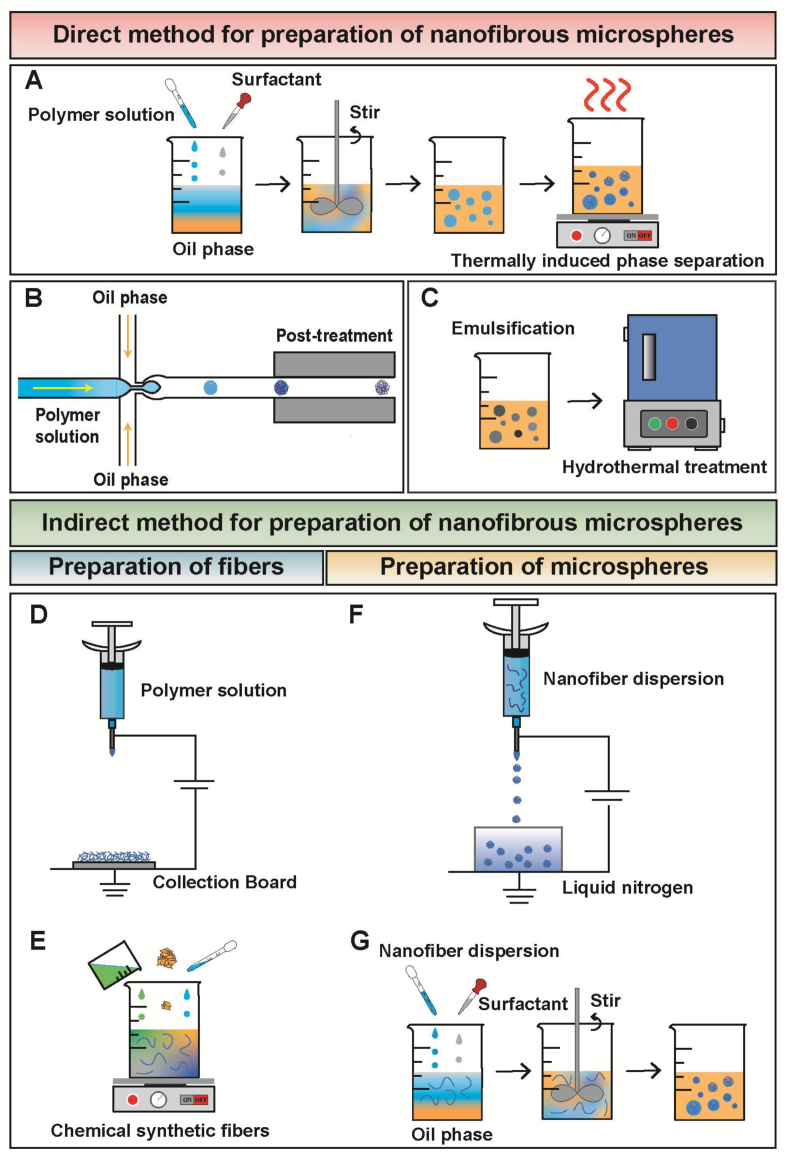
Direct method and indirect method for nanofibrous microsphere fabrication.

So far, a variety of methods have been used for the preparation of nanofibrous microspheres [34]. Basing on the formation sequence of microsphere and nanofiber, herein, we classify the fabrication techniques of nanofibrous microspheres into two types: direct method and indirect method (Table 1). For direct method, microsphere formation occurs prior to nanofiber network development. Specifically, microspheres are prepared first by emulsification (Fig. 2A) [35], and microfluidics (Fig. 2B) [37], etc, to serve as templates. Subsequently, nanofibers form in situ and assemble into nanofibrous networks with/without post-treatments [42], such as thermally induced phase separation (TIPS) [36], and hydrothermal treatment (Fig. 2C) [38]. Direct method features mild reaction condition, simple fabrication procedure and high controllability over microsphere microstructure and size. However, materials suitable for direct method are restricted to a small group of biomaterials, such as gelatin, chitosan, bacterial cellulose, PLA and TiO_2_, etc. In addition, for direct method, preparation of microsphere templates through emulsion holds great potential for industrial production, but suffers from poor particle size uniformity. On the contrary, preparation of microsphere templates using microfluidics allows to obtain microspheres with high uniformity, but suffers from low throughput and limited mass production potential. Indirect methods, on the contrary, normally starts with preparation of nanofibers through electrospinning (Fig. 2D), chemical synthesis (Fig. 2E), etc. The nanofibers are then suspended and homogenized in aqueous solution. Upon obtaining a homogeneous nanofiber suspension, the nanofibers self-assemble into microspheres through various methods including electrospray + freeze drying (Fig. 2F) [43], emulsification + freeze drying [44], and emulsification + thermally induced phase separation [45] (Fig. 2G), etc. Indirect method is applicable to the vast majority of biomaterials, whether they are natural or synthetic, organic or inorganic. Indirect method allows precise control over nanofiber diameter, nanofiber length, sphere size, pore size and pore structure [46]. Nevertheless, it is hard to produce nanofibers through electrospinning or chemical synthesis in a large scale. Moreover, the multi-step process of indirect method increases fabrication complexity and cost. Despite considerable progress has been achieved in the field of nanofibrous microsphere fabrication, development of nanofibrous microspheres with controllable size and physical and biochemical property, high uniformity and scale-up production potential is still challenging, which we believe must be overcome in order to move nanofibrous microspheres from bench to bedside.

**Table 1:** Comparison of direct and indirect methods.

Method	Nanofiber formation method	Sphere formation method	Materials	Structural controllability	Process complexity	Yield	Cost	Scalability	Reference
Direct method	Thermally induced phase separation	Emulsion method	Most of natural polymers and their derivative materials (gelatin, chitin, bacterial cellulose, etc.)	Tunable sphere size and nanofiber diameter	Simple	High	Low	High throughput; Poor size uniformity	[35,36]
	Physical/chemical treatment	Microfluidic method	Chitosan, polylactic acid (PLA), gelatin methacryloyl (GelMA)	Controllable sphere size and nanofiber diameter	Relatively complex	Low	Moderate	Low large-scale production potential	[37]
	Hydrothermal treatment	Emulsion method	TiO_2_	Controllable sphere size and surface topography	Relatively complex	Moderate	High	Limited to heat-resistant materials	[38]
Indirect method	Electrostatic spinning	Electrostatic spray	Polycaprolactone (PCL), gelatin, GelMA, etc.	Controllable nanofiber diameter, sphere size and pore structure	Relatively complex	Moderate	Moderate	Limited large-scale production potential	[39]
	Chemical treatment/synthesis	Electrostatic spray	Silk protein, GelMA	Controllable sphere size	Relatively complex	High	Moderate	Limited large-scale production potential	[40]
	Chemical synthesis	Emulsion method	Cellulose/Polyvinyl alcohol (PVA)	Controllable sphere size and morphology	Relatively complex	High	Moderate	Limited large-scale production potential	[41]

## 3 Research progress of nanofibrous microspheres made from diverse biomaterials

Biomaterials, such as natural biopolymers, synthetic biopolymers and inorganic biomaterials, exhibit excellent biocompatibility, thereby have been widely used to prepare nanofibrous microspheres for biomedical applications including bone regeneration [[47], [48], [49]], cartilage regeneration [45], vascular regeneration [50,51], dental treatment [[52], [53], [54]] and skin repair [55]. Different fabrication strategies using diverse kinds of raw materials produce nanofibrous microspheres with distinct characteristics varying in composition, topography, pore size, porosity, and stiffness [29,56]. Based on the chemical composition of nanofibers, herein, we classify nanofibrous microspheres into five types, namely, natural polymer-based (polymers obtained by isolation and purification of natural biomaterials, e.g., chitin, cellulose, collagen, silk fibroin) nanofibrous microspheres, natural polymer-derived (polymers obtained by isolation, purification and physicochemical treatment of natural biomaterials, e.g., gelatin, chitosan, GelMA) nanofibrous microspheres, synthetic polymer-based (e.g., poly(L-lactic acid) (PLLA), poly(lactic-co-glycolic acid) (PLGA)) nanofibrous microspheres, inorganic nanofibrous microspheres and composite nanofibrous microspheres. In this section, we will summarize recent progress of nanofibrous microspheres made from different biomaterials, with a focus on their design principle, microstructure, bioactivity and biomedical applications.

### 3.1 Natural polymer-based nanofibrous microspheres

Natural polymers, widely found in nature, are high-molecular-weight compounds formed through crosslinking of repeated monomers [57]. Polysaccharide (e.g., chitin, cellulose), protein (e.g. silk fibroin, collagen), rubber, and resin are typical representatives of natural polymers, among which, polysaccharides and proteins are widely used in drug delivery, tissue engineering, and regenerative medicine due to their excellent biocompatibility and/or biodegradability. So far, a variety of polysaccharide- and protein-based nanofibrous microspheres have been developed. In this part, we will introduce the progress of polysaccharide- and protein-based nanofibrous microspheres.

#### 3.1.1 Natural polysaccharide-based nanofibrous microspheres

**Fig. 3:**
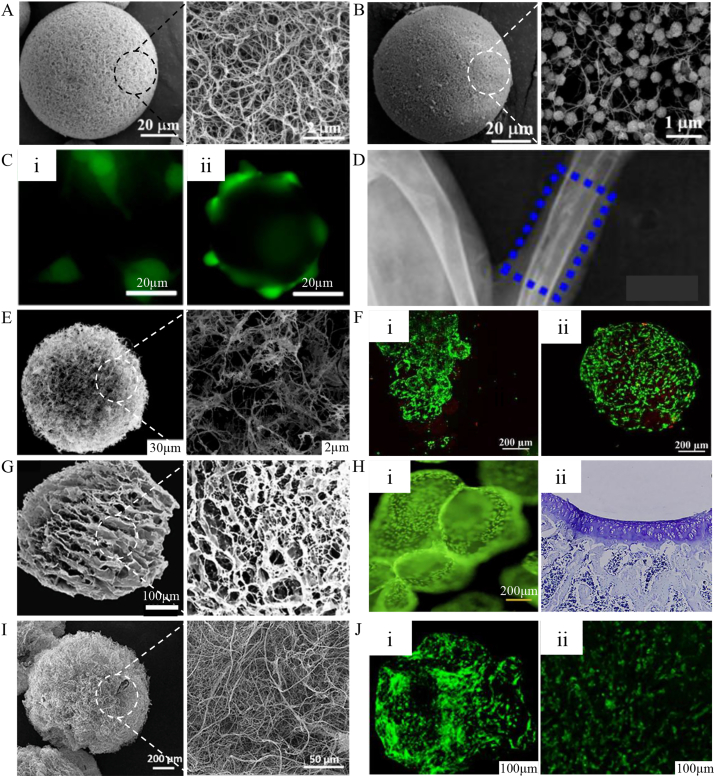
SEM images of chitin nanofibrous microspheres (A) and hydroxyapatite/chitin nanofibrous microspheres. (C) Live staining of MC3T3-E1 cells cultured on chitin nanofibrous microspheres (i) and hydroxyapatite/chitin nanofibrous microspheres (ii). (D) X-ray examination of bone defect treated with hydroxyapatite/chitin nanofibrous microspheres for 12 weeks. Reproduced with permission from Ref. [58], Copyright 2017, ACS Publications. (E) SEM images of cellulose nanofibrous microspheres. Reproduced with permission from Ref. [59], Copyright 2014, ACS Publications. (F) Live/dead staining of 3T3 cells cultured on cellulose nanofibrous microspheres with diameter of 94.5 μm (i) and 503.9 μm (ii) for 10 days, respectively. Reproduced with permission from Ref. [41], Copyright 2017, Springer. (G) SEM images of DBC nanofibrous microspheres with 66.7% DHYL-DBC mass ratio. (H) Confocal microscope image of microtissues (i) and toluidine blue staining of repaired cartilage (ii). Reproduced with permission from Ref. [45], Copyright 2018, Elsevier. (I) SEM images of silk fibroin nanofibrous microspheres. (J) Live/dead staining of BMSCs cultured on silk fibroin nanofibrous microspheres (i) and conventional silk fibroin sponges (ii) at day 7. Reproduced with permission from Ref. [40], Copyright 2022, ACS Publications. (For interpretation of the references to colour in this figure legend, the reader is referred to the Web version of this article.)

Chitin is a polysaccharide extracted from the shells of marine crustaceans, featuring low toxicity, good biocompatibility, high mechanical strength and low cost. Duan et al. synthesized chitin nanofibrous microspheres through a thermally induced self-assembly method [36]. The diameter of the chitin nanofibers and the size of the chitin nanofibrous microspheres were tunable to be in the range from 26 to 55 nm and 3 to 130 μm, respectively, by adjusting thermal induction temperature and varying processing parameters including oil/water ratio, surfactant amount, and stirring speed. The chitin nanofibrous microspheres were highly porous with pore size of several micrometers, supporting immortal normal human hepatic cell line L02 cell attachment with high efficiency. Nevertheless, the L02 cells exhibited round shape with poor spreading on the microspheres, indicating limited cell affinity of the chitin nanofibrous microspheres. A step further, the same group developed chitin nanofiber/hydroxyapatite (HA) microspheres by using chitin nanofibrous microspheres as templates for in situ growth of HA crystals (Fig. 3A–B) [58]. Due to the presence of HA crystals, the bioactivity of the chitin nanofibrous microspheres was significantly improved including promoted cell adhesion and enhanced in vivo bone healing (Fig. 3C–D).

Cellulose, composed of glucose, is the main component of plant cell walls and is the most abundant polysaccharide in nature. It is biocompatible and low cost, thereby has been explored for biomedical applications including tissue engineering, wound dressing, and drug delivery. For example, Cai et al. prepared cellulose nanofibrils through mechanical defibrillation of commercial bleached softwood kraft pulp and fabricated cellulose nanofibrous microspheres by a spray-freeze-drying method (Fig. 3E) [59]. The nanofibrous microspheres were highly porous with pore size ranging from nano-to micrometers, endowing these microspheres with remarkable water uptake capacity and ultralow density. In addition, these covalently cross-linked microspheres were very stable even in a harsh environment. Finally, they supported the attachment and proliferation of NIH3T3 cells, indicating their potential as cell culture scaffold. In 2017, Zhang et al. proposed a method combining emulsification process with freeze-drying to produce cellulose nanofibrous microspheres [41]. Polyvinyl alcohol was used as binder to stabilize the nanofibrous microspheres. The cellulose nanofibrous microspheres possessed a highly porous structure. Compared with small cellulose nanofibrous microspheres (∼94.5 μm), large cellulose nanofibrous microspheres (∼503.9 μm) seemed to provide a more favorable environment for 3T3 cells to attach to and proliferate upon (Fig. 3F), which was probably attributed to they had proper pore size for the growth of 3T3 cells. In 2018, Wang et al. prepared dialdehyde bacterial cellulose (DBC) nanofibrous microspheres through emulsification method (Fig. 3G) [45]. DL-allo-hydroxylysine (DHYL) was in-situ grafted on the DBC fibers to improve the biocompatibility of DBC by introduction of amino acids. DHYL-DBC was then mixed with chitosan to form DBC nanofibrous microsphere. The pore size, porosity, mechanical properties, and biodegradation rate of the nanofibrous microsphere was tunable by adjusting the ratio between chitosan and DHYL-DBC. Bone marrow mesenchymal stem cells (BMSCs) cultured on nanofibrous microsphere showed improved proliferation compared with those cultured on chitosan microcarriers, which could be partially attributed to the fact that the nanofibrous architecture of the DBC nanofibrous microspheres possessing high surface area could absorb cell adhesion proteins at high levels. After three weeks of co-culture, BMSCs and DBC nanofibrous microspheres turned into functional microtissues (Fig. 3Hi). Upon implantation into a knee articular cartilage defect in mice, the microtissues facilitated cartilage repair dramatically (Fig. 3Hii).

Chitin and cellulose are quite abundant in nature. Nevertheless, their bioactivity is relatively low. Modification and combination with other bioactive materials may be an effective strategy to improve the bioactivity of these polysaccharide-based natural polymer nanofibrous microspheres, thereby enhancing their application potential.

#### 3.1.2 Natural protein-based nanofibrous microspheres

Collagen is the most abundant protein in the human body, possessing excellent biocompatibility, biodegradability, and bioactivity. So far, collagen has been widely used in biomedical fields. However, to the best of our knowledge, preparation of collagen nanofibrous microspheres has been rarely reported except for Chan's work. Chan et al. fabricated collagen microspheres with nanofibrous network structure trough an emulsification method combined with a self-assembled collagen fiber reconstitution process [60]. The collagen nanofibrous microspheres were consisted of randomly orientated nano-size collagen fibers (around 45 nm in diameter) with open meshwork. Although the application of the collagen nanofibrous microspheres was not intended for cell carrier but for protein drug delivery, the fabrication strategy provides a reference for the production of collagen nanofibrous microspheres.

Silk fibroin is a natural protein extracted from silkworm cocoons, featuring high biocompatibility, biodegradability, low immunogenicity, and adjustable mechanical stability, making it widely used in biomedical field. Yan et al. developed silk fibroin nanofibrous microspheres by using silk fibroin nanofibers as building blocks. The silk fibroin nanofibers were exfoliated from natural silks by chemical pretreatment and mechanical disintegration, and then assembled into microspheres (Fig. 3I) [40]. The silk fibroin nanofibrous microspheres were highly porous with a high specific surface area. Compared with silk fibroin sponge without fibrous topography, silk fibroin nanofibrous microspheres showed superior performance on promoting proliferation (Fig. 3J) and osteogenic differentiation of MSCs, suggesting their potential as cell carriers and tissue engineering microscaffolds.

Natural protein-based nanofibrous microspheres share high homology with human native components, thereby possessing superior bioactivity than those of polysaccharide nanofibrous microspheres. However, they have the disadvantages of potential immunogenicity and high processing and manufacturing cost.

### 3.2 Natural polymer-derived nanofibrous microspheres

Natural polymer derivatives, such as gelatin, GelMA, chitosan and carboxymethyl chitosan, are biomaterials obtained through physicochemical treatment of collagen and chitin [24]. On the one hand, they exhibit outstanding biocompatibility comparable to those of their natural counterparts. On the other hand, they possess additional properties including thermosensitivity, photosensitivity, water solubility and antibacterial property, etc, attributed to the physicochemical treatment. Therefore, natural polymer derivatives have been widely used for nanofibrous microsphere fabrication.

#### 3.2.1 Natural polysaccharide-derived nanofibrous microspheres

**Fig. 4:**
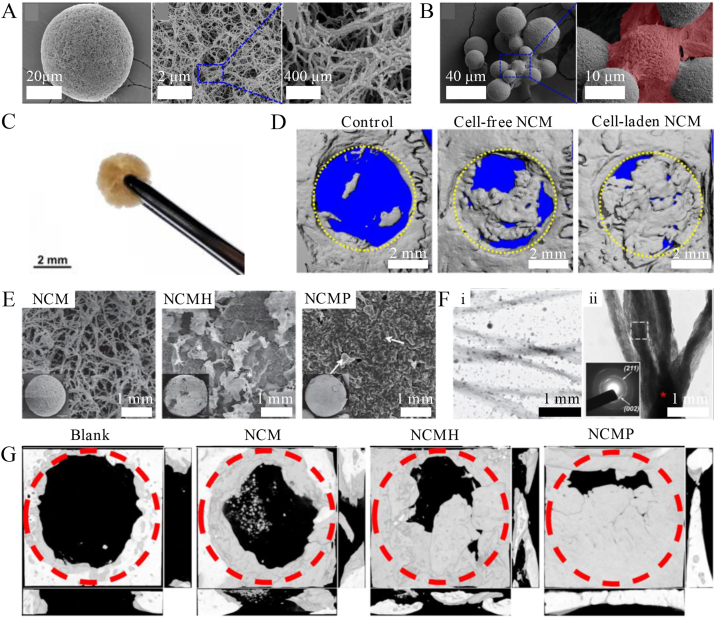
(A) SEM images of NCM. (B) SEM images of MC3T3-E1 cells cultured on NCM after 3 days, cells were rendered in red. (C) Digital photograph of an in vitro macroscopic 3D cell-NCM constructs. (D) Micro CT reconstruction images of bone defect. Reproduced with permission from Ref. [62], Copyright 2022, Elsevier. (E) SEM images of NCM, HAp@NCM (NCMH) and PAH-ACP@NCM (NCMP). (F) TEM images of single-layer collagen with NCMP at 3 days (i) and 5 days (ii). (G) 3D reconstruction images of bone samples treated with NCM, NCMH, and NCMP at 8 weeks. Reproduced with permission from Ref. [63], Copyright 2025, Elsevier. (For interpretation of the references to colour in this figure legend, the reader is referred to the Web version of this article.)

Chitosan is a natural linear polysaccharide obtained by deacetylation of chitin, possessing good biocompatibility, solubility, antibacterial property and hemostatic capability. In 2016, Zhou et al. prepared chitosan microspheres with an ECM-mimicking nanofibrous structure through alkaline induced direct gelation of chitosan microsphere emulsions [61]. The diameter of the chitosan nanofibers as well as the chitosan nanofibrous microspheres could be tuned by adjusting the concentration of neutralizing agent and the manufacturing parameters of microfluidic devices. Due to the presence of ECM-mimicking nanofibrous structure, the chitosan nanofibrous microspheres could provide more effective anchoring sites for cell attachment and proliferation than solid chitosan microspheres. Upon co-culture of two weeks, a macroscopic 3D geometrically shaped cartilage-like composite could be constructed with the chitosan nanofibrous microspheres as bottom-up cell-carrier building blocks, suggesting the application potential of the chitosan nanofibrous microspheres as cell-carrier components for cartilage tissue engineering. However, chondrocytes showed limited spreading on the chitosan nanofibrous microspheres and hardly infiltrated into the nanofibrous structure, which might be attributed to the hydrophobicity and relatively poor bioactivity of chitosan. In 2022, Yang et al. prepared nanofibrous chitosan microspheres (NCM) via thermal induction of chitosan molecular chain from alkaline/urea aqueous solution (Fig. 4A) [62]. The chitosan nanofibrous microspheres showed ECM-mimicking microstructure. Cells could recognize topographical characteristics through contact guidance. Upon culture with NCM, MC3T3-E1 cells attached, proliferated, and colonized on the NCM (Fig. 4B), transforming loose microspheres into a macroscopic millimeter-sized cell-NCM constructs (Fig. 4C). RT-qPCR test showed that the expression level of adhesion-related genes including ITGA5, ITGB1, FAK, PXN and VCL of MC3T3-E1 cells on NCM was significantly higher than that of MC3T3-E1 cells on solid chitosan microspheres. Activation of focal adhesion and actin cytoskeleton signaling pathway genes (FAK, PXN, and VCL) could promote focal adhesion formation, regulate cytoskeletal organization, and advance cell spreading. After implantation in rat calvarial defects, rat mesenchymal stem cells (RMSCs)-laden NCM constructs enhanced the healing of the defects, resulting in a larger amount of new bone (Fig. 4D). In 2025, the same group developed a bio-delivery system by modifying the NCM with poly (allylamine hydrochloride)-stabilized amorphous calcium phosphate (PAH-ACP) to mediate mineralization during bone regeneration (Fig. 4E) [63]. The PAH-ACP@NCM (NCMP) could function similarly to matrix vesicles by allowing PAH-ACP released from NCMP to retain its amorphous state, thereby infiltrating and mineralizing collagen fibrils (Fig. 4F). The presence of PAH-ACP up-regulated the expression of cell adhesion-related genes and osteogenic-related genes expression, reduced inflammatory responses, and promoted in situ biomineralization in rat calvarial defects (Fig. 4G). All in all, chitosan nanofibrous microspheres are relatively easy to fabricate. Nevertheless, their bioactivity is relatively low. Although they could be modified with other biomaterials to attain enhanced bioactivity to some extent, their clinical translation as cell-carrier and tissue engineering building blocks is still challenging.

#### 3.2.2 Natural protein-derived nanofibrous microspheres

**Fig. 5:**
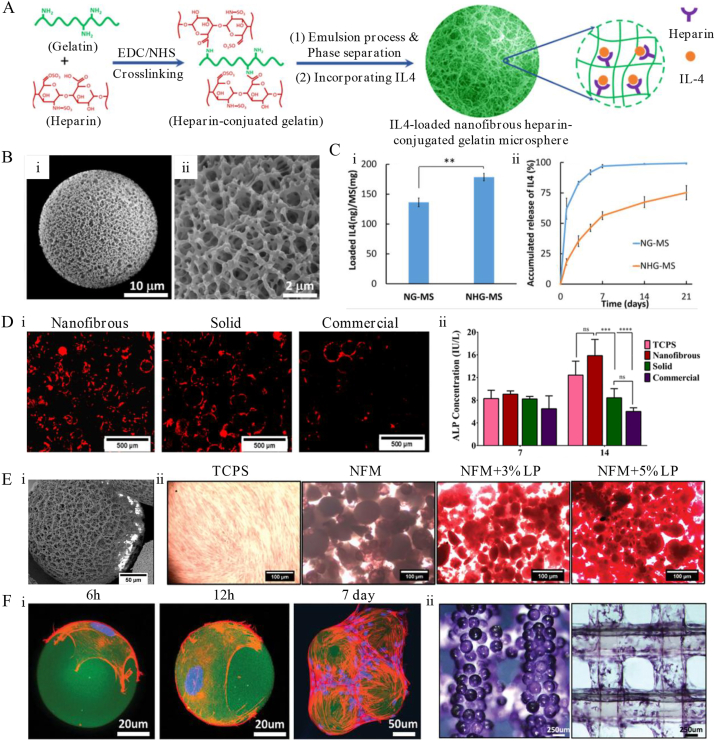
(A) Schematic illustration of preparation of IL4-loaded NHG-MS. (B) SEM images of NHG-MS. (C) Loading efficiency (i) and release profiles (ii) of IL4 from nanofibrous heparin-modified gelatin microsphere (NHG-MS) and nanofibrous gelatin microsphere (NG-MS). Reproduced with permission from Ref. [64], Copyright 2017, ACS Publications. (D) Fluorescence microscopy images (i) and ALP expression (ii) of BMSCs cultured for 7 days on gelatin microspheres with different surface topography. Reproduced with permission from Ref. [65], Copyright 2022, Elsevier. (E) SEM image (i) of laponite-loaded nanofibrous microspheres (NFM + LP) and optical images of mineralized matrixes formed by human dental follicle stem cells cultured for 21 days on gelatin nanofibrous microspheres (NFM) and NFM + LP. Reproduced with permission from Ref. [66], Copyright 2022, Wiley. (F) Confocal 3D reconstruction images (i) showing the adhesion and spreading of BMSCs cultured for 6 h, 12 h, and 7 days on GelMA nanofibrous microspheres, and ALP staining images (ii) of BMSCs on GelMA nanofibrous microspheres scaffolds and conventional GelMA scaffolds after 7 days culture. Reproduced with permission from Ref. [67], Copyright 2023, Wiley.

Gelatin, a protein derived from partial hydrolysis of collagen extracted from the connective tissues, skin, and bones of animals, exhibits remarkable biocompatibility and biodegradability. In addition, gelatin possesses varying free chains (hydrogen-bound α, β, γ chains), water solubility, low immunogenicity, large crosslinking functional groups and unique thermos-sensitive behavior, making it a preferred choice of polymer for biomedical applications. In 2017, Hu et al. fabricated heparin-modified gelatin nanofibrous microsphere (NHG-MS) by combining water/oil emulsification technique and thermally induced phase separation process (Fig. 5A–B) [64]. The nanofibrous microstructure endowed the NHS-MS with high interleukin 4 (IL4) loading efficiency, low density and high porosity (Fig. 5C). The NHS-MS could release IL4 in a sustained manner, modulate macrophage polarization, attenuate inflammatory responses and promote osteogenic differentiation and bone regeneration. In 2022, Sarviya et al. employed an microemulsion coupled with TIPS method to fabricate gelatin nanofibrous microspheres with variable physicochemical properties such as size, surface properties, surface chemistry, and degradability [65]. Due to the nanofiber and porous architecture mimicking 3D ECM microenvironment, the gelatin nanofibrous microspheres exhibited superior performance on facilitating stem cell binding (Fig. 5Di), enhancing stem cell proliferation, and inducing stem cell differentiation such as osteogenic and chondrogenic lineages (Fig. 5Dii), compared to solid gelatin microspheres. All these characteristics indicate that the gelatin nanofibrous microspheres may serve as a promising candidate for ex-vivo cells' expansion and injectable carriers for stem cell transplantation. In addition, the physicochemical properties and bioactivity of the gelatin nanofibrous microspheres could be further modulated by incorporating other materials into the nanofibrous network. For instance, introducing laponite into the gelatin nanofibrous network to develop laponite-gelatin nanofibrous microspheres (Fig. 5Ei) [66]. The incorporation of laponite significantly enhanced the osteogenic inducing properties of the nanofibrous microspheres, resulting in higher ALP activity, osteogenic gene expression levels and significant alizarin red staining (Fig. 5Eii). In 2023, Hu et al. conjugated gelatin nanofiber with EPLQLKM (E7) peptide to develop E7-conjugated gelatin nanofibrous microspheres [68]. E7 peptide selectively promotes bone marrow-derived BMSCs and periodontal ligament stem cells (PDLSCs) adhesion but inhibits the attachment and spreading of epithelial cells and gingival fibroblasts. The E7-conjugated gelatin nanofibrous microspheres is highly porous (93.2% porosity), degradable and injectable. As a biological barrier, the E7-conjugated gelatin nanofibrous microspheres facilitated BMSCs to occupy the periodontal defect, leading to significant improvements in periodontal regeneration. Based on that, the same group further developed E7-conjugated gelatin nanofibrous hollow microspheres [69]. Bone morphology protein 7-loaded calcium phosphate nanoparticles were encapsulated in the hollow space of the nanofibrous hollow microspheres to enhance the osteogenesis of BMSCs. The size of the nanofibrous hollow microspheres was around 50 μm. The morphology and structure of the nanofibrous hollow microspheres could be adjusted by varying gelatin concentration, volume of inner oil, and stirring time. Due to the presence of E7, bone morphology protein 7 and calcium phosphate nanoparticles, the gelatin nanofibrous hollow microspheres significantly enhanced alveolar bone tissue regeneration in a rat fenestration defect model.

GelMA is a photosensitive biopolymer obtained by methacryloyl modification of gelatin. In addition to good biocompatibility and biodegradability, GelMA exhibits unique photocrosslinking capability. Upon irradiation by UV light, GelMA solution could solidify quickly and transform into hydrogel with stable three-dimensional network [70]. In 2024, Hu et al. synthesized GelMA nanofiber microspheres by integrating microfluidics with thermally induced phase separation techniques [67]. The size of the GelMA nanofiber microspheres could be tailored by adjusting flow rate ratio between continuous phase (GelMA solution) and dispersed phase (mineral oil), and the nanostructure and porosity of the GelMA nanofiber microspheres could be modulated by varying GelMA concentrations. The GelMA nanofiber microspheres supported adhesion and spreading of BMSCs (Fig. 5Fi). In addition, the GelMA nanofiber microspheres could be printed into 3D scaffolds. GelMA nanofibrous microsphere scaffolds possessed interconnected microporous network and high surface area, which could provide sufficient adhesive sites and ample space for cell attachment, migration and proliferation, and enhance the diffusion of oxygen and nutrients. Therefore, BMSCs exhibited more pronounced proliferation and infiltration on GelMA nanofibrous microsphere scaffolds than those on conventional GelMA hydrogel scaffolds with smooth surface. Moreover, BMSCs showed much higher expression of osteogenic genes and ALP and significantly more calcium and collagen deposition on GelMA nanofibrous microsphere scaffolds than those on conventional GelMA hydrogel scaffolds (Fig. 5Fii). Nevertheless, the underlying mechanism was not investigated. In vivo, the GelMA nanofibrous microsphere scaffolds facilitated much more new bone formation than conventional GelMA hydrogel scaffolds. The author proposed that the hierarchical porous structure of the GelMA nanofibrous microsphere scaffolds could facilitate the influx of initial immune cells and the exchange of bioactive factors, thereby resulting in a moderate immune response and a pro-regenerative immune microenvironment. The porous structure could also enhance the survival of loaded exogenous stem cells, facilitate the diffusion of their secreted bioactive factors into defect area, and promote the secretion and deposition of collagen and minerals. All these effects orchestrated concurrently, leading to superior bone regeneration.

Taken together, protein-derived nanofibrous microspheres provide both ECM-mimic microstructure and composition. Their bioactivity is normally superior to that of polysaccharide-derived nanofibrous microspheres. They could be used as drug carrier, cell carrier or foundational building block for 3D printing, thereby holding great versatility for tissue engineering application.

### 3.3 Synthetic polymer nanofibrous microspheres

**Fig. 6:**
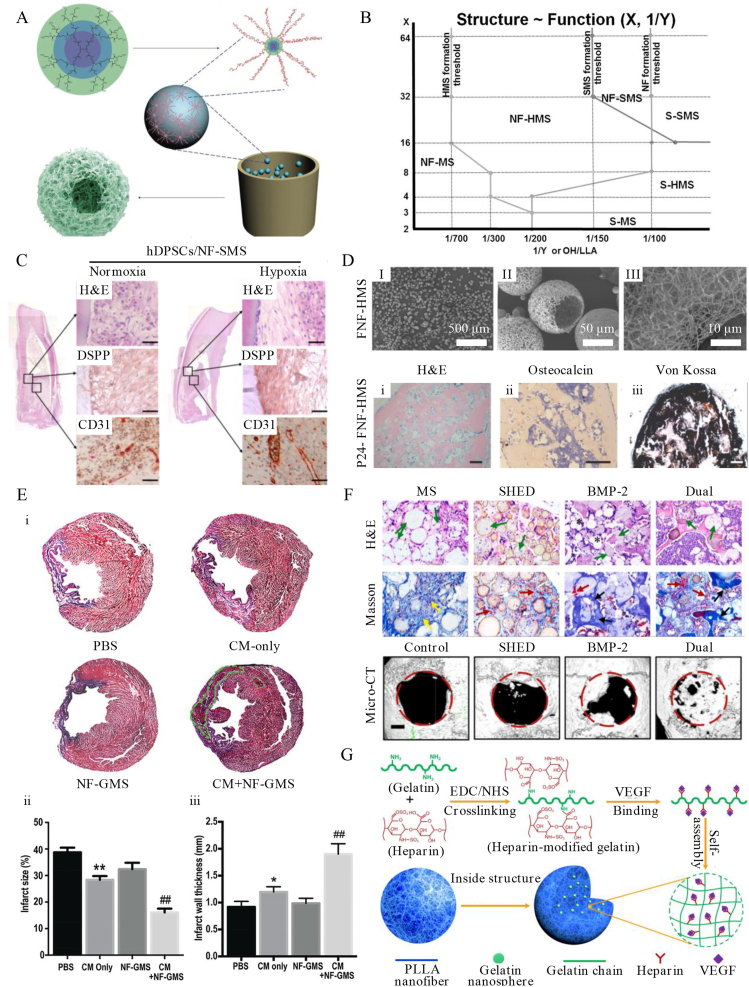
(A) Schematic illustration of SS-PLLA synthesis and nanofibrous hollow microsphere fabrication. Reproduced with permission from Ref. [74], Copyright 2011, Springer. (B) The structure of SS-PLLA microspheres as a function of arm number and arm length of SS-PLLA. Reproduced with permission from Ref. [75], Copyright 2015, Wiley. (C) Pulp tissue regeneration enhanced by hypoxia-primed hDPSCs/SS-PLLA nanofibrous microspheres in a subcutaneous tooth implantation model. Scale bars: 50 μm. Reproduced with permission from Ref. [76], Copyright 2016, Elsevier. (D) SEM micrographs (I,II,III) of PLLA-g-PHEMA functional nanofibrous hollow microspheres (FNF-HMS) and histological analysis (i,ii,iii) after 5 week mouse subcutaneous injection of BMP2-mimic P24 conjugated FNF-HMS (P24-FNF-HMS) with rabbit BMSCs. Scale bar: 100 μm. Reproduced with permission from Ref. [77], Copyright 2014, Wiley. (E) Masson trichrome staining (i), quantitative analysis (ii), and wall thickness (iii) of rat myocardial infarction hearts treated with PBS, cardiomyocyte (CM), PLLA-PEG-PNIPAm nanofibrous gelling microspheres (NF-GMS) and CM-loaded NF-GMS for 4 weeks' transplantation. Reproduced with permission from Ref. [78], Copyright 2020, Wiley. (F) H&E staining and Masson's trichrome staining of ectopic implants using PDA-NF-MS and Hep-Dopa NF-MS (ratio is 1:1, MS group), SHED adhered on PDA-NF-MS (SHED group), rhBMP-2 chemically bonded to Hep-Dopa NF-Ms (BMP-2 group), and combination of SHED/PDA-NF-MS and BMP-2/Hep-Dopa NF-Ms (ratio is 1:1, Dual group), and reconstructive 3D micro-CT photographs of repaired cranial bone defects in Control (blank), SHED, BMP-2, Dual group at 8 weeks post-operation. Scale bar: 1 mm. Reproduced with permission from Ref. [79], Copyright 2019, Elsevier. (G) Schematic illustration of synthesis of VEGF-loaded gelatin nanospheres@PLLA nanofibrous microspheres. Reproduced with permission from Ref. [80], Copyright 2016, Elsevier.

Synthetic polymers, such as PLLA [71,72], PCL and PLGA [73], are artificially synthesized macromolecular polymers formed through polymerization of repeating monomer units. Compared with natural biopolymers, synthetic biopolymers possess more customizable molecular weight, chemical composition and physical structure. Moreover, they have low immunogenicity and no endotoxin. Since 2011, Ma group has developed a series of polylactic acid-based nanofiberous microspheres as cell and drug carrier for tissue repair. For instance, in 2011, they synthesized star-shaped poly(L-lactic acid) (SS-PLLA) nanofibrous hollow microsphere through a surfactant-free emulsification process (Fig. 6A) [74]. The nanofibrous hollow microspheres were composed of nanofibers with an average diameter of 160 ± 67 nm. The size of the nanofibrous hollow microspheres could be controlled from a few micrometers to a few hundred micrometers by adjusting stirring speed and SS-PLLA concentration. The nanofibrous hollow microspheres exhibited high porosity up to 96.7%. Compared with solid PLLA microspheres with smooth surface, nanofibrous hollow SS-PLLA microspheres showed higher cell attachment efficiency since nanofibrous structure might adsorb cell adhesion proteins (such as fibronectin and vitronectin) at higher levels than smooth surface. Chondrocytes cultured on nanofibrous hollow SS-PLLA microspheres exhibited much higher proliferation rate and expressed much more glycosaminoglycans (GAG) than those on solid PLLA microspheres. Subcutaneous pocket injection into nude mice showed chondrocyte-loaded nanofibrous hollow SS-PLLA microsphere group exhibited higher of GAG/wet-weight (w/w) and GAG/DNA ratios compared to those of chondrocyte-loaded solid PLLA microsphere group and chondrocyte alone group. The author suggested that the high surface areas of the nanofibrous hollow microspheres probably enhanced protein adsorption for cell-scaffold interactions and facilitated mass transfer for tissue regeneration. The faster degradation rate of the nanofibrous hollow microspheres and their hollow structure probably provided additional space for matrix accumulation, facilitating cartilage tissue formation. Subsequent study showed that the number and length of SS-PLLA arms significantly influenced the porous structure of the SS-PLLA nanofibrous microspheres (Fig. 6B) [75]. In addition to chondrocyte, the SS-PLLA nanofibrous microspheres were shown to be able to deliver human dental pulp stem cells (hDPSCs) and promote dental pulp tissue regeneration (Fig. 6C) [76]. In 2015, they introduced poly(hydroxyethyl methacrylate) (PHEMA) blocks to PLLA and designed PLLA-based graft copolymers poly(l-lactic acid)-graft-poly(hydroxyethyl methacrylate) (PLLA-g-PHEMA) hollow nanofibrous microspheres (Fig. 6D) [77]. The PLLA-g-PHEMA hollow nanofibrous microspheres could effectively deliver cytomodulin and bone morphogenetic protein-2, inducing chondrogenesis for cartilage formation and ectopic bone formation in nude mice (Fig. 6D). In 2020, they reported a poly(l-lactic acid)-b-poly(ethylene glycol)-b-poly(N-Isopropylacrylamide) copolymer (PLLA-PEG-PNIPAm) which could self-assemble into nanofibrous microspheres. These nanofibrous microspheres were thermally responsive, which could be injected and then transform into 3D hydrogel after injection in vivo triggered by physiological temperature(Fig. 6E) [78]. Compared to direct injection of cardiomyocytes, transplantation of cell-laden PLLA-PEG-PNIPAm nanofibrous microspheres significantly reduced infarct size, enhanced graft integration, stimulated vascularization in the infarct zone, leading to a substantial recovery of cardiac function. Instead of design of PLLA-based graft copolymers, some researchers tried to modulate the physiological properties of PLLA nanofibrous microspheres via surface modification. For instance, Fang et al. used polydopamine (PDA) and heparin-dopamine (Hep-Dopa) to modify PLLA nanofibrous microspheres [79]. PDA-modified PLLA nanofibrous microspheres (PDA-NF-MS) facilitated spreading of stem cells from human exfoliated deciduous teeth (SHED) and supported retention of SHED over 3 weeks in vivo. Hep-Dopa-modified PLLA nanofibrous microspheres (Hep-Dopa NF-MS) exhibited higher recombinant human bone morphogenic protein-2 (rhBMP-2) loading efficiency and better rhBMP-2 release property. Upon implantation together, the rhBMP-2-loaded Hep-Dopa PLLA nanofibrous microspheres and the SHED-loaded PDA-modified PLLA nanofibrous microspheres significantly promoted bone tissue regeneration in both ectopic and orthotopic site (Fig. 6F). In addition to surface modification, the same group adjusted the drug delivery property of PLLA nanofibrous microsphere by immobilizing VEGF-loaded gelatin nanospheres in the nanofibers of the PLLA nanofibrous microspheres (Fig. 6G) [80]. These microspheres supported the adhesion of DPSCs and released VEGF in a sustained manner, allowing targeted co-delivery of stem cells and therapeutic molecules for dental pulp regeneration. Besides gelatin nanospheres, mesoporous silica nanoparticles, drug loaded PLGA nanospheres [81], and γ-Fe_2_O_3_ nanoparticles [82] have also been tried to incorporate into PLLA nanofibrous microsphere system.

Besides PLA, PCL has also been used for the fabrication of nanofibrous microspheres. For example, Chen et al. fabricated PCL nanofibrous microspheres by electrospinning and electrospray techniques [39]. Bioglass particles were incorporated into the PCL nanofibers to improve their bioactivity. The diameter of the PCL fibers was around 1 μm and the diameter of the PCL nanofibrous microspheres was tunable ranging from 1 to 5 mm by varying electrospray voltage. The PCL nanofibrous microspheres supported BMSCs attachment, proliferation and promoted BMSCs osteogenic differentiation in vitro. In vivo, BMSCs loaded PCL nanofibrous microspheres showed substantial improvements in both angiogenesis and osteogenic differentiation, as well as promoting the healing of bone defects.

In addition to PLLA and PCL, there are also many other types of synthetic polymers, such as PLGA, polyurethane (PU), and poly(trimethylene carbonate) (PTMC), etc. PLGA possesses adjustable biodegradability. The degradation rate of PLGA could be tuned by adjusting the ratio of lactic acid to glycolic acid. PLGA nanofibers can be easily obtained through electrospinning, while fabrication of nanofibrous microspheres by using pure PLGA nanofibers as building blocks has been rarely reported. There are a few studies reported fabrication of composite nanofibrous microspheres by using PLGA/protein composite nanofibers, which will be discussed in the following section. PU nanofibers and PTMC nanofibers could also be obtained by electrospinning. Some studies reported development of PU nanofibrous membranes [83] and PTMC nanofibrous membranes [84] for tissue repair. Considering their outstanding mechanical property and biocompatibility, we believe PU and PTMC are promising candidates for manufacturing new nanofibrous microspheres.

To conclude, synthetic polymer nanofibrous microspheres has the advantage of customizable composition and structure. They have low immunogenicity and high biocompatibility. Although their bioactivity is hardly comparable to that of protein nanofibrous microspheres, they could obtain enhanced biocompatibility through post treatment including surface modification and incorporation of functional nanoparticles, thereby serving as a promising candidate for cell and drug delivery.

### 3.4 Inorganic nanofibrous microspheres

**Fig. 7:**
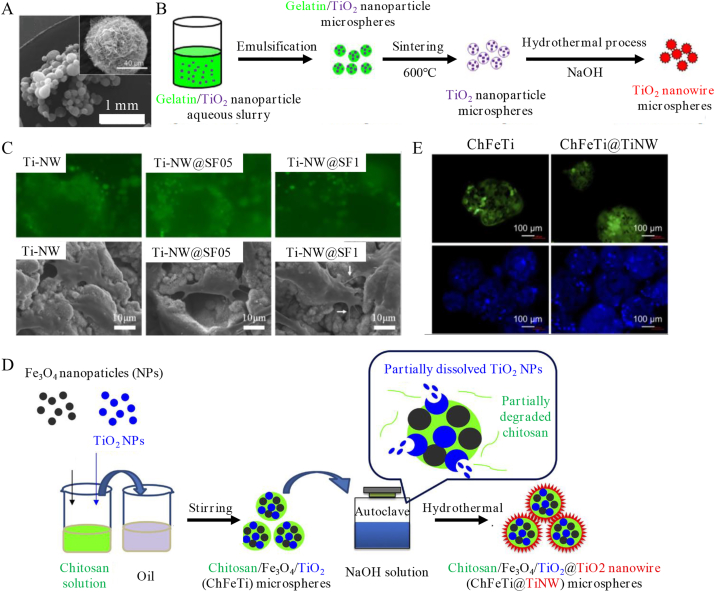
(A) SEM image of bioglass nanofibrous microspheres. Reproduced with permission from Ref. [43], Copyright 2018, ACS Publications. (B) Schematic illustration for synthesis of silk fibroin-decorated TiO2 nanowire microspheres. (C) Live/dead staining and SEM images of fibroblast L929 cells cultured on TiO2 nanowire microspheres and silk fibroin-decorated TiO2 nanowire microspheres. Reproduced with permission from Ref. [85], Copyright 2022, Elsevier. (D) Schematic illustration of synthesis of chitosan/Fe_3_O_4_/TiO_2_@TiO_2_ nanofibrous microspheres. (E) Live/dead staining, and nuclei staining of HUVECs cultured on chitosan/Fe_3_O_4_/TiO_2_@TiO_2_ microspheres (ChFeTi) and chitosan/Fe_3_O_4_/TiO_2_@TiO_2_ nanofibrous microspheres (ChFeTi@TiNW). Reproduced with permission from Ref. [86], Copyright 2023, Wiley.

Inorganic biomaterials, such as calcium phosphate and bioglass, possess chemical composition similar to that of the inorganic part of natural bone tissue, thus exhibiting excellent biocompatibility and bioactivity. So far, inorganic biomaterials have been widely used for drug delivery, tissue repair and tissue regeneration in the form of 3D printed scaffolds, injectable cements, nanoparticles and films, etc. Compared with polymer nanofibrous microspheres, reports of fabrication of inorganic nanofibrous microspheres are fewer. In 2018, Boda et al. fabricated bioglass nanofibrous microspheres by electrospinning of bioglass nanofibers and subsequent assembly of these nanofibers into microspheres through electrospray (Fig. 7A) [43]. In 2022, Zhang et al. reported TiO_2_ nanowire microspheres (Fig. 7B) [85]. TiO_2_ microspheres were synthesized first by emulsification and sintering. The microspheres were then hydrothermally treated in NaOH solution to obtain TiO_2_ nanowire microspheres. The TiO_2_ nanowire microspheres were further decorated with silk fibroin to enhance their biocompatibility. In vitro, the silk fibroin-decorated TiO_2_ nanowire microspheres showed enhanced cell spreading and proliferation due to the presence of silk fibroin (Fig. 7C). In 2023, Bai et al. fabricated nanofibrous and hollow titania microspheres through in situ alkali hydrothermal treatment of hollow titania microspheres [38]. Typically, hollow titania microspheres were synthesized using emulsified chitosan/gelatin microspheres as sacrificial templates via a sol-gel route. Hollow titania microspheres were then hydrothermally treated in a concentrated NaOH solution to obtain nanofibrous and hollow titania microspheres. Compared with non-fibrous titania microspheres, nanofibrous titania microspheres exhibited enhanced cell attachment and proliferation. In addition, the nanofibrous titania microspheres could strongly adsorb tetracycline hydrochloride and release the drug in a sustained manner due to the presence of the nanofibrous and hollow structure. The same group also developed chitosan/Fe_3_O_4_/TiO_2_@TiO_2_ nanofibrous microspheres using similar hydrothermal treatment method(Fig. 7D) [86]. TiO_2_ nanowires on the surface of the microspheres contributed to a hierarchical and nanofibrous architecture and an improved specific surface area. The nanofibrous microspheres significantly enhanced the attachment and proliferation of human umbilical vein endothelial cells (HUVECs) compared with non-fibrous microspheres (Fig. 7E), which might be attributed to TiO_2_ nanowires could provide more anchorage sites for cell attachment and proliferation.

Apart from above-mentioned inorganic nanofibrous microspheres, to the best of our knowledge, almost no other inorganic nanofibrous microspheres have been reported so far. Nevertheless, a variety of inorganic nanofibers have been successfully synthesized to date, such as hydroxyapatite nanowire [87], magnesium oxide nanowire [88], and calcium silicate nanowire [89], etc. These inorganic nanofibers possess good biocompatibility, high aspect ratio and structural flexibility which are especially suitable for constructing self-supporting three-dimensional network structures and mechanical reinforcement. For instance, Zhu et al. reported synthesis of hydroxyapatite nanowires with high aspect ratio and excellent flexibility [87]. By using hydroxyapatite nanowires as building blocks, they developed highly flexible and nonflammable inorganic hydroxyapatite paper and ultralight inorganic hydroxyapatite aerogel [90,91]. Wang et al. synthesized calcium silicate nanowires and used them as reinforcement material to fabricate composite hydrogel with enhanced mechanical property and bioactivity [92]. Regarding the good biocompatibility and bioactivity, high aspect ratio and structural flexibility of above-mentioned inorganic nanofibers, we believe design new kind of inorganic nanofibrous microspheres by using these inorganic nanofibers as building blocks, such as calcium phosphate nanofibrous microspheres and calcium silicate nanofibrous microspheres, may provide inspiration for development of advanced cell carriers for tissue regeneration, especially hard tissue regeneration.

### 3.5 Composite nanofibrous microspheres

**Fig. 8:**
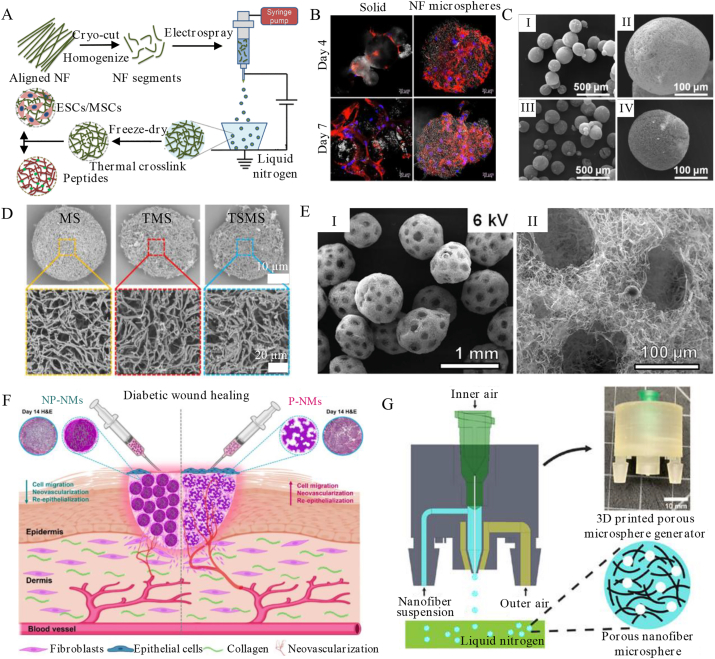
(A) Schematic overview of fabrication of composite nanofibrous microspheres and their applications for stem cell and drug/peptide delivery. (B) Confocal images of rBMSCs cultured on PCL-gelatin solid microspheres and PCL-gelatin nanofibrous microspheres for 4 and 7 days. Reproduced with permission from Ref. [43], Copyright 2018, ACS Publications. (C) SEM images showing nanofibrous microspheres composed of PCL:gelatin:GelMA (1:0.5:0.5) nanofiber segments (I, II) before and (III, IV) after crosslinking. Reproduced with permission from Ref. [93], Copyright 2019, Elsevier. (D) SEM micrographs of gelatin/PLA-based nanofiber microspheres (MS), tannic acid-modified nanofiber microspheres (TMS) and tannic acid/Sr^2+^-modified nanofiber microspheres (TSMS). Reproduced with permission from Ref. [94], Copyright 2023, Elsevier. (E) SEM micrographs showing nanofibrous microspheres with controlled open macropores. Reproduced with permission from Ref. [46], Copyright 2020, Wiley. (F) Potential mechanism of enhanced healing by injectable porous PLGA-gelatin composite nanofibrous microspheres at the diabetic mouse wound site. Reproduced with permission from Ref. [55], Copyright 2024, ACS Publications. (G) Schematic illustrating the fabrication procedures of porous composite nanofibrous microspheres using a 3D-printed microfluidic platform. Reproduced with permission from Ref. [95], Copyright 2025, Wiley.

Synthetic biopolymers, such as PLA, PLGA and PCL, normally exhibit poor bioactivity due to the fact that they lack cell recognition sites. However, they are strong in mechanical property. On the contrary, protein possesses good bioactivity but exhibits poor mechanical strength. Therefore, composite nanofibrous microspheres consisting of synthetic biopolymer/protein composite nanofiber building blocks have been developed aiming to develop nanofibrous microspheres that combines the advantage of synthetic biopolymer and proteins. For instance, in 2018, Boda et al. fabricated a variety of composite nanofibrous microspheres by combining electrospinning and electrospray [43]. Typically, they first prepared PCL-gelatin nanofibers and PLGA-gelatin nanofibers by electrospinning, which were then homogenized to form uniform nanofiber dispersions. Subsequently, composite nanofibrous microspheres were obtained by electrospray of these nanofiber dispersions into liquid nitrogen followed by freeze-drying and thermal treatment (Fig. 8A). The size and structure of the nanofibrous microspheres could be tuned by adjusting electrospray voltage, nanofiber concentration, etc. Compared with conventional solid microspheres, these nanofibrous microspheres elicited enhanced proliferation and differentiation of rBMSCs (Fig. 8B). Similar method has been used to prepare BMP-2 peptide-conjugated/VEGF-mimicking QK peptide-conjugated PCL-gelatin-GelMA nanofibrous microspheres [93] (Fig. 8C) and metal phenolic networks-modified PLA-gelatin nanofibrous microspheres [94] (Fig. 8D). In 2020, Xie group reported a gas bubble–mediated coaxial electrospray method to prepare composite nanofibrous microspheres (e.g. PCL-gelatin, PCL-gelatin-GelMA and PLGA-collagen-gelatin) with controlled open macropores (Fig. 8E) [46]. The pore number and pore size were controlled by monitoring airflow rate. Compared to nonporous composite nanofibrous microspheres, open porous composite nanofibrous microspheres supported human neural progenitor cell growth in 3D with a larger number and more neurites. In addition, porous composite nanofibrous microspheres showed faster cell infiltration and host tissue integration than nonporous composite nanofibrous microspheres after subcutaneous injection to rats. In 2024, they used the same method to prepare porous PLGA-gelatin composite nanofibrous microspheres for diabetic wound healing [55]. Compared to nonporous nanofibrous microspheres, porous PLGA-gelatin nanofibrous microspheres supported better cell infiltration, and showed superior cell protection performance. In vivo, the porous PLGA-gelatin composite nanofibrous microspheres promoted host cell infiltration, neovascularization, and re-epithelialization in a diabetic mouse wound model (Fig. 8F), implying their effectiveness in healing diabetic wounds. In 2025, the same group presented a 3D-printed microfluidic platform that could be used for large-scale fabrication of porous composite nanofibrous microspheres with precise control over size, pore architecture, and morphology (Fig. 8G) [95].

Taken together, composite nanofibrous microspheres combine the advantage of synthetic biopolymers and proteins. On the one hand, they exhibit good biocompatibility and bioactivity due to the presence of protein component. On the other hand, they possess outstanding mechanical property that surpasses conventional polysaccharide and protein nanofibrous microspheres. In addition, the fabrication method of these composite nanofibrous microspheres is relatively easy, thus holding possibility for realization of mass production and clinical translation.

A detailed comparison regarding the composition, advantage and disadvantage, as well as application direction of different nanofibrous microsphere systems is shown in Table 2.

**Table 2:** Comparison of various nanofibrous microspheres.

Category	Material	Cost	Hydrophilicity/Hydrophobicity	Biodegradability	Bioactivity	Application	Reference
Natural polymer-based nanofibrous microspheres	Polysaccharides: chitin, cellulose; Proteins: collagen, silk fibroin	Polysaccharides: low cost; Proteins: high cost	Chitin, cellulose: hydrophobic; Protein: hydrophilic	Chitin, cellulose: Hardly degradable; Protein: degradable	Chitin, cellulose: low (can be enhanced by modification); Proteins: high	Bone/cartilage regeneration, blood purification	[36,40,41,45,[58], [59], [60]]
Natural polymer-derived nanofibrous microspheres	Polysaccharide derivatives: chitosan; Protein derivatives: gelatin, GelMA	Moderate	Chitosan: hydrophobic; Protein: hydrophilic	Chitosan: Hardly degradable; Protein: degradable	Polysaccharide derivatives: relatively low; Protein derivatives: high	Cartilage/bone regeneration, Guided tissue regeneration	[[61], [62], [63], [64], [65], [66], [67], [68], [69], [70]]
Synthetic polymer-based nanofibrous microspheres	PLLA、PCL	High	Hydrophobic	Degradable	Low (can be enhanced by modification)	Knee repair, pulp regeneration, cartilage/bone regeneration, heart regeneration	[39,[74], [75], [76], [77], [78], [79], [80], [81], [82]^83^]
Inorganic nanofibrous microspheres	Bioglass、TiO_2_	Moderate/High	Hydrophilic	Bioglass: degradable; TiO_2_: non-degradable	Bioglass: high; TiO_2_: low	Bone repair	[38,43,85,86]
Composite nanofibrous microspheres	PCL-gelatin, PLGA-gelatin, PLA-gelatin, PCL-gelatin-GelMA	Moderate/High	Moderately hydrophilic	Degradable	High	Bone repair, diabetic wound healing, osteoarthritis treatment	[43,46,55,[93], [94], [95]]

## 4 Potential mechanism of nanofibrous microspheres influence stem cell behavior

In recent years, studies have demonstrated that microenvironment plays a crucial role in regulating stem cell behavior [96]. Different kinds of nanofibrous microspheres exhibit varied stiffness, topography, porosity, pore size, and dimension. Tremendous evidence have shown that stem cells could sense this kind of physical signals via integrin-mediated signaling, focal adhesion kinase (FAK) pathways, and Yes-associated protein (YAP)/transcriptional co-activator with PDZ-binding motif (TAZ) mechanotransduction, etc, and respond accordingly, such as through adhesion, migration, proliferation, and differentiation [97].

Transmembrane receptors, primarily integrins, can sense the stiffness of ECM and transfer this signal from ECM to cytoskeleton structures via integrin-mediated cell-ECM adhesion (focal adhesion, FA). Following initial focal complex formation, mechanosensitive proteins, such as talin, paxillin and vinculin are activated. Activation of these proteins further modulate adhesion signaling, strengthen or weaken the linkage between adhesion complexes and actin, thereby influencing cell adhesion [98]. In addition to cytoskeleton structures, the signal can be further transmitted into nucleus via FA–actomyosin cytoskeleton–linker of nucleoskeleton and cytoskeleton complex–SUN proteins–lamins. Stiffness-sensitive nuclear lamins undergo stretch or contraction, which in turn causes nuclear pores to deform. Stretched nuclear pores facilitate the entry of transcriptional regulators, such as YAP and TAZ, into the nucleus, thus affecting YAP/TAZ localization and activity, gene expression, and cell differentiation [99,100]. Normally, soft substrates enhance β1 integrin internalization via caveolae/raft-dependent endocytosis, which blocks BMP/Smad signaling and promotes MSCs neurogenic differentiation. Substrates with intermediate stiffness drive actin filament bundling and stress fiber contraction through integrin β3–RhoA–Rho-associated kinase (ROCK)-myosin light chain kinase pathway, generating appropriate cytoskeletal tension that contributes to myogenic gene expression. Stiff substrates increase RUNX2 activity through integrin α2-ROCK-FAK-ERK1/2 pathway and promotes MSCs osteogenic differentiation. Moreover, substrate stiffness can also regulate Cyclin D1 (a cell cycle promoter) expression through FAK, ERK, and Rac signaling pathways, thereby affecting cell cycle progression and proliferation [101].

In terms of topography, stem cells have been shown to be able to sense the characteristics of nanofiberous materials including fiber diameter and fiber arrangement. For instance, neural stem cells tended to differentiate into neurons on fibers with small diameter, and into glial cells on fibers with large diameter [102]. MSCs cultured on PLLA nanofibers with a diameter of 580 nm showed enhanced osteogenic differentiation compared to those cultured on PLLA nanofibers with a diameter of 1210 nm. The author speculated that BMP/SMAD signaling pathway might be involved in this diameter-dependent effect [103]. In addition to differentiation, nanofiber diameter could also influence stem cell migration. For example, nanofibers with a diameter of 400 nm were shown to be more conducive to MSCs migration than those with a diameter of 800 nm/1200 nm [104]. Regarding fiber arrangement, some studies have reported that oriented arrangement of nanofibers induced the oriented arrangement of stem cells. Compared with stem cells that were randomly distributed, highly aligned stem cells exhibited higher proliferation and migration rate, and higher osteogenic differentiation tendency [103,105,106] which might be attributed to the elevated expression of mechanosensive proteins including integrin, FAK and ROCK [107,108].

Porosity and pore size can also influence stem cell behavior. Porous structures provide conducive environment for oxygen and nutrients supply as well as waste removal, facilitating stem cell proliferation. Compared with small pores, large pores are superior in supporting stem cell infiltration. Furthermore, it has been reported that pore architecture affects stem cell differentiation [109]. Nevertheless, the specific signaling pathways involved in this process still needs further investigation. Regarding microsphere size, the size of nanofibrous nanospheres used as stem cell carriers normally ranges from tens of microns to hundreds of microns [110]. Small microspheres are more suitable for injection and minimally invasive treatment, while large microspheres could provide larger interstitial gap for cell migration and infiltration when transplanted in vivo [67]. Microsphere size may impact stem cell behavior by influencing the spatial distribution of cytoskeletal force. Microspheres with different sizes have different curvatures. It has been shown that substrate curvature affects the size and shape of stem cell actin stress fibers, FAs, and nuclei, thereby resulting in a significant influence on cell adhesion processes [111]. However, how microsphere size/curvature affects stem cell proliferation and differentiation has been rarely reported so far, and the underlying mechanism still needs further investigation.

In summary, stem cells could sense the physical characteristics of nanofibrous microspheres through various mechanisms, making it possible for researchers to program stem cell behavior by designing nanofibrous microspheres with proper stiffness, topography, porosity, pore size, and dimension.

## 5 Conclusion and outlook

In this review, we highlighted recent advances in development of nanofibrous microspheres with diverse composition, size, morphology and structure by using a variety of methods for regeneration medicine applications. Based on the sequence of microsphere formation and nanofiber formation, we classified the fabrication method of nanofibrous microspheres into direct method and indirect method. Different fabrication method features distinct advantage, such as precise size control, elaborate structure modulation, or high large-scale production potential. Further, we divided current nanofibrous microspheres into five types, e.g., natural polymer-based nanofibrous microspheres, natural polymer-derived nanofibrous microspheres, synthetic polymer-based nanofibrous microspheres, inorganic nanofibrous microspheres, and composite nanofibrous microspheres according to the chemical composition of their nanofiber building blocks. Each kind of nanofibrous microspheres has its own characteristics. For instance, polysaccharide nanofibrous microspheres are abundant in raw material resources. Protein nanofibrous microspheres share high homology with human native ECM. Synthetic polymer nanofibrous microspheres feature customizable mechanical property, chemical composition and biodegradability. Inorganic nanofibrous microspheres hold great potential for hard tissue repair application. Composite nanofibrous microspheres combine the advantage of synthetic biopolymers and proteins.

### 5.1 Clinical translation challenges

A lot studies have demonstrated that nanofibrous microspheres mimicking natural ECM microstructure are superior in facilitating cell attachment, spreading, proliferation and up-regulating expression of specific genes, such chondrogenic and osteogenic genes, compared to non-fibrous microspheres. However, despite considerable progress that has been achieved in developing different methods for nanofibrous microsphere fabrication and designing different kinds of nanofibrous microsphere with diverse composition and structure, there are still limitations and challenges need to be addressed in order to move nanofibrous microspheres from bench to bedside. Firstly, what are the optimal structural parameters (e.g., size, modulus, nanofiber diameter) of nanofibrous microsphere carriers is not clear. It is important to understand how these structural parameters influence stem cell behavior and tissue repair process synergistically. Secondly, GMP production of nanofibrous microspheres with uniform size in a large scale is still challenging. Thirdly, quality standard and regulatory compliance regarding the size, size uniformity, nanofiber diameter, biocompatibility, biodegradability, endotoxin content, immune response, and long-term in vivo biosafety of nanofibrous microspheres need to be established. We believe that addressing these questions will bring current basic studies a step closer to clinical applications.

### 5.2 Future research perspective

Current nanofibrous microspheres are predominantly limited to polymeric materials, which suffer from uncontrolled swelling, high hydrophobicity that impedes cell attachment and spreading (e.g., synthetic polymer, chitin, and chitosan), and high risk of endotoxin contamination (e.g., animal-derived protein). Therefore, develop new nanofibrous microspheres, such as fully inorganic bioglass and bioceramic nanofibrous microspheres with anti-swelling ability, low endotoxin contamination risk, high cell affinity, and investigate their drug/cell delivery as well as tissue regeneration potential will be of significant value.

The structural parameters of nanofibrous microspheres are highly adjustable, such as porosity, pore size, sphere size, sphere modulus, and nanofiber diameter, etc. In addition, these parameters are highly correlated; a change in one parameter often causes a change in another. Each of these parameters could influence stem cell behavior. Therefore, it seems impossible to decipher what kind of combination of these parameters would be optimal for stem cells by using conventional methods. In the past few years, artificial intelligence (AI) has emerged as a powerful tool for data processing, structure prediction, and biomaterial design. AI models built upon massive amounts of data and advanced algorithms possess excellent analytical and reasoning capabilities. Therefore, combining AI model with conventional experimental technique might be a promising research direction to find out the optimal structural parameters of nanofibrous microspheres.

Regarding their ECM-like microstructure and outstanding cell loading capability, we speculate nanofibrous microspheres might be able to serve as an ideal stem cell carrier for exosome production by providing stem cells with a microenvironment similar to that of natural cell niche. In addition, we believe nanofibrous microspheres may serve as promising building blocks for the construction of heterogeneous artificial tissues/organs due to their advantages of small size and easy assembly. Last but not least, development of smart nanofibrous microspheres with enhanced/controlled therapeutic effects [112], e.g, environmentally responsive nanofibrous microspheres that could accelerate tissue regeneration in response to external stimuli including magnetic field [113], electric field, and acoustic signal, etc, would be an interesting topic.

## CRediT authorship contribution statement

**Tengfei Tian:** Conceptualization, Investigation, Methodology, Writing – original draft. **Jiashan Zhang:** Investigation, Methodology. **Jiahui Wu:** Investigation, Methodology. **Chuanfeng An:** Conceptualization, Funding acquisition, Writing – review & editing. **Huanan Wang:** Conceptualization, Methodology, Writing – review & editing. **Yonggang Zhang:** Conceptualization, Investigation, Methodology, Writing – original draft, Writing – review & editing.

## Declaration of competing interest

The authors declare that they have no known competing interests.

## Data Availability

The data that has been used is confidential.
